# Cognitive, behavioral, and autonomic correlates of mind wandering and perseverative cognition in major depression

**DOI:** 10.3389/fnins.2014.00433

**Published:** 2015-01-05

**Authors:** Cristina Ottaviani, Leila Shahabi, Mika Tarvainen, Ian Cook, Michelle Abrams, David Shapiro

**Affiliations:** ^1^IRCCS Santa Lucia FoundationRome, Italy; ^2^ENPlab, Department of Psychology, Sapienza University of RomeRome, Italy; ^3^Department of Medicine, University of California, Los AngelesLos Angeles, CA, USA; ^4^Department of Applied Physics, University of Eastern FinlandKuopio, Finland; ^5^Department of Clinical Physiology and Nuclear Medicine, Kuopio University HospitalKuopio, Finland; ^6^Department of Psychiatry and Biobehavioral Sciences, Semel Institute for Neuroscience and Human Behavior, David Geffen School of Medicine, University of California, Los AngelesLos Angeles CA, USA

**Keywords:** perseverative cognition, rumination, mind wandering, heart rate, heart rate variability, ecological momentary assessment, ambulatory monitoring, major depression disorder

## Abstract

Autonomic dysregulation has been hypothesized to play a role in the relationships between psychopathology and cardiovascular risk. An important transdiagnostic factor that has been associated with autonomic dysfunction is perseverative cognition (PC), mainly present in Major Depressive Disorder (MDD) in the form of rumination. As the ability to adaptively let our mind wander without ruminating is critical to mental health, this study aimed to examine the autonomic concomitants of functional vs. dysfunctional intrusive thoughts in MDD. Ambulatory heart rate (HR) and variability (HRV) of 18 MDD subjects and 18 healthy controls were recorded for 24 h. Approximately every 30 min during waking hours subjects reported their ongoing thoughts and moods using electronic diaries. Random regression models were performed. Compared to controls, MDD subjects were more often caught during episodes of PC. In both groups, PC required more effort to be inhibited and interfered more with ongoing activities compared to mind wandering (MW) (*ps* < 0.0001). This cognitive rigidity was mirrored by autonomic inflexibility, as PC was characterized by lower HRV (*p* < 0.0001) compared to MW. A worse mood was reported by MDD patients compared to controls, independently of their ongoing cognitive process. Controls, however, showed the highest mood worsening during PC compared to being on task and MW. HRV during rumination correlated with self-reported somatic symptoms on the same day and several dispositional traits. MDD subjects showed lower HRV during sleep, which correlated with hopelessness rumination. Results show that PC is associated with autonomic dysfunctions in both healthy and MDD subjects. Understanding when spontaneous thought is adaptive and when it is not may clarify its role in the etiology of mood disorders, shedding light on the still unexplained association between psychopathology, chronic stress, and risk for health.

## Introduction

In the past decades, a growing interest in difficulties staying “in the moment” as a hallmark of Major Depressive Disorder (MDD) has rapidly developed. The relationship between the tendency of the mind to wander and depression has been hypothesized to be bi-directional. Several studies provide evidence of mind wandering as a consequence of depressive state. For example, Smallwood et al. (2009) experimentally manipulated mood and showed that negative relative to positive mood reduced the amount of attentional commitment to the task probably by enhancing the focus on task irrelevant personal concerns. Poerio et al. (2013) used a 7-day experience sampling technique showing that sadness tended to precede mind wandering but mind wandering itself was not associated with later mood. Other authors, however, suggest that mind wandering may be not only a consequence but also a cause of low mood. Killingsworth and Gilbert (2010) analyzed experience samples from 2250 participants concluding that “a wandering mind is an unhappy mind.” Stawarczyk et al. (2013) provided further evidence for the involvement of mind wandering in predicting subsequent levels of momentary negative affect. Moreover, several studies suggest an association between dysphoria and enhanced mind wandering. Murphy et al. (2013) supported this hypothesis by manipulating the occurrence of mind wandering in participants with varying levels of self-reported dysphoria. Smallwood et al. (2007) showed that in dysphoric individuals mind wandering led to greater decoupling from task-relevant processing and greater physiological arousal, as indexed by higher heart rate (HR).

Consistent with these results, interventions aimed to enhance control over mind wandering, such as mindfulness-based cognitive therapy (MBCT; Teasdale, 1999) have been shown to be effective in preventing relapses in depression (Williams and Kuyken, 2012). Also, training aimed at increasing executive control (e.g., Siegle et al., 2014) or working memory capacity (Onraedt and Koster, 2014) have been shown to be beneficial in reducing a well-established antecedent for depression, i.e., ruminative thinking. In fact, by reducing mind wandering, these therapeutic approaches are effective because they target the ideal context for rumination to occur (e.g., Marchetti et al., 2014), the latter being a vulnerability factor for the onset, maintenance, and recurrence of MDD (e.g., Nolen-Hoeksema et al., 2008). Contrary to what happens during depressive rumination, mind wandering can facilitate a series of positive outcomes, such as creative problem solving, prospection, or relief from monotonous and boring activities (see Smallwood and Schooler, 2014 for a recent review).

In the present study, we aimed at investigating if mind wandering *per se* is associated with a series of maladaptive consequences, such as mood worsening and autonomic imbalance or if it does so only when it takes the form of rumination about the past or worry about the future. To the best of our knowledge, this question was directly addressed only in a previous laboratory study that we conducted on a sample of healthy participants (Ottaviani et al., 2013). Here, our main purpose is to further investigate this issue in patients with a diagnosis of MDD in a more ecological setting. Ecological momentary assessment has been suggested as the best way to study depression: first, it helps avoiding the recall bias toward negative memories that characterizes MDD; second, it facilitates generalization to real life; third, combining it with simultaneous physiological assessment may ultimately help explain why MDD patients are at increased risk for cardiovascular disease, and finally, insights obtained from this type of assessment may not only serve researchers and clinicians, but may also benefit patients directly (reviewed in Aan het Rot et al., 2012).

A few studies investigated mind wandering with the use of ecological momentary assessment (McVay et al., 2009; Killingsworth and Gilbert, 2010; Song and Wang, 2012; Unsworth et al., 2012; Carriere et al., 2013), but only in healthy individuals and none of them simultaneously recorded physiological activity. Conversely, this has been widely done to study the physiological consequences of rumination and worry (Brosschot et al., 2007; Pieper et al., 2007, 2010; Slatcher et al., 2010; Ottaviani et al., 2011; Weise et al., 2013). However, existing studies focused only on healthy individuals. Overall, results show a sustained physiological activation across several biological systems in association with rumination and worry. The few studies that have been conducted in MDD patients combining ecological momentary assessment with concomitant physiological recording have so far mostly focused on cortisol. Together, results point toward a dysfunction of the hypothalamic-pituitary-adrenal system in responding to daily hassles in MDD patients (Peeters et al., 2003a, 2004). Huffziger et al. (2013) found reduced mood-cortisol coupling in remitted recurrent depression, suggesting that during the course of recurrent depression hypothalamic-pituitary-adrenal axis activation may become less responsive to subtle emotional experiences in natural setting. Another ecological study of MDD patients measured prefrontal brain activity using electroencephalography (EEG) at baseline and subsequent rumination during the following week (Putnam and McSweeney, 2008). Results showed that only in MDD patients and not in healthy controls, less prefrontal neural activity was associated with higher rumination and lower self-esteem.

To our knowledge, this is the first study to combine ecological momentary assessment with simultaneous ambulatory HR and variability (HRV) recording in MDD. This is surprising considering that patients suffering from MDD show profound autonomic dysfunctions characterized by a decrease in HRV (e.g., Udupa et al., 2007; Koschke et al., 2009; Wang et al., 2013). Moreover, HRV has been found to be a significant predictor of treatment response and remission from MDD (Chambers and Allen, 2002; Jain et al., 2014), and HRV biofeedback seems a promising tool for the treatment of MDD (Karavidas et al., 2007).

Based on our previous findings in healthy individuals (Ottaviani et al., 2013), we hypothesized that mind wandering fails to serve its adaptive function, and turns into a risk factor for health whenever it becomes rigid and inflexible. As ruminative and worrisome thoughts show different content but no differences in appraisals and strategies (Watkins et al., 2005), we collapsed them into one category under the umbrella term of perseverative cognition (PC) (see Brosschot et al., 2010). We expect that, both in MDD and healthy subjects, PC would be associated with higher levels of cognitive (higher efforts to inhibit), behavioral (interference with ongoing activities), and autonomic (lower HRV) inflexibility compared to mind wandering and being focused on a task. Based on our hypothesis that mind wandering is not *per se* maladaptive but becomes dysfunctional only when it takes the form of PC, we expect only the latter to be associated with mood worsening. In light of the role of vagal functioning as a hallmark of psychological and somatic health (Thayer and Lane, 2009), we expect to find an association between HRV during PC and psychopathological dispositions or somatic complaints on the same day. Finally, we hypothesized these effects to be potentiated in MDD patients compared to controls.

## Materials and methods

### Participants

The sample was composed of 18 subjects [12 women, 6 men; mean age = 38.4 (12.1) years] who met diagnostic criteria for a current major depressive episode and 18 healthy controls [11 women, 7 men; mean age = 30.1 (10.5) years], 13 Asian, 13 Caucasian, 3 African, and 7 Latino Americans. Participants were recruited by the use of flyers and participation in previous studies. Exclusionary criteria were: being younger than 18, a diagnosis of psychotic or personality disorders, current active suicidal ideation or intent, a diagnosis of heart disease or other serious medical illness, use of drugs/medications that might affect HR and HRV, obesity (body mass index > 32 kg/m^2^), or pregnancy. Participants were compensated ($35) for their time. The experimental protocol was approved by the UCLA Institutional Review Board.

### Procedure

After a pre-screening phone interview to rule out exclusionary criteria, participants came to the lab, read and signed the informed consent form, and underwent either the Structured Clinical Interview for DSM-IV (SCID) or the Mini International Neuropsychiatric Interview (M.I.N.I), administered by a trained clinician to confirm the diagnosis of MDD. If eligible, participants filled out the online questionnaires and were then instructed about the cell phone used for the electronic diary and the ambulatory HR device. The ambulatory device was attached to their chest, and they left the laboratory. After approximately 24 h, participants were asked to return the ambulatory device and the phone. On that occasion, they completed a post-assessment online questionnaire (data not presented here), were debriefed, and received monetary compensation.

### Questionnaires

During the first laboratory session, participants completed on line a series of socio-demographic (age and sex) and lifestyle (nicotine, alcohol, and caffeine consumption, physical exercise) questions and questionnaires to measure levels of: (a) hostility (CM; Cook and Medley, 1954), (b) anger (STAXI; Spielberger et al., 1985), (c) trait anxiety (STAI-X2; Spielberger et al., 1970), (d) depression (PHQ-9; Kroenke et al., 2001 and BDI; Beck et al., 1996), and (e) Loneliness (Three-Item Loneliness Scale; Hughes et al., 2004). Three forms of PC were assessed: (1) depressive rumination (RRS; Nolen-Hoeksema and Morrow, 1991), measured by how often people engage in responses to depressed mood that are self-focused (e.g., I think “Why do I react this way?”), symptom-focused (e.g., I think about how hard it is to concentrate), and focused on the possible consequences and causes of one's mood (e.g., I think “I won't be able to do my job if I don't snap out of this”); (2) worry (PSWQ; Meyer et al., 1990), that is mainly focused on future outcomes (e.g., “As soon as I finish one task, I start to worry about everything else I have to do”) and therefore prevalent in anxiety disorders but also present in MDD; and (3) reactive rumination (SRRS; Robinson and Alloy, 2003), as a measure of the tendency to ruminate after the occurrence of stressful events (i.e., not confounded with depressive symptoms). The SRRS has three subscales: Negative Inferential Style “Ruminate about how the stressor will affect other areas of your life,” Hopelessness “Think about the possibility that things will never get better,” and Active Problem-Solving “Try to find something positive in the situation or something you've learned.”

### Electronic diary

Participants were provided with an electronic diary implemented on an Android phone. At variable times (about every 30 min), the phone alarm signaled participants to complete the diary. Each diary asked them to report: (1) what they were thinking about (About what you are doing, About a problem you have, About a past experience, About something that may happen in the future, Planning something, About someone you know, About your feelings or mood, Having a passing thought, Other); (2) the duration of the thought (5 min, 10 min, 20 min or since the last beep); (3) if the thought was Positive, Negative, or Neutral; (4) if the thought was repetitive (from 0 = Not at all to 4 = Very much); (5) the temporal dimension of the thought (Past, Future, Present); (6) if the thought was interfering with what they were doing (from 0 = Not at all to 4 = Very much); (7) how much they were trying to suppress the thought (from 0 = Not at all to 4 = Very much); (8) if they had experienced one or more annoying or disturbing events in the preceding period (Yes/No); (9) information on factors that may affect HR, including posture, physical activity, and food, caffeine, nicotine, and alcohol consumption since the last diary report; and (10) their current levels of feeling Tired, Anxious, Sad, Happy, Angry, and Bored using a 5-point scale from Not at all to Very much. The diary included questions about social interaction but these data go beyond our present aim and will not be presented. Based on their content, valence, temporal dimension, and repetitiveness, thoughts were re-coded as: being on task, mind wandering (MW), and ruminating or worrying (PC). The exact coding flowchart is available from the first author, upon request. By using the touch screen, responses could be made in a few minutes. Before bedtime, subjects were asked to fill out the Patient Health Questionnaire (PHQ-15 for somatization; Kroenke et al., 2002) and, upon awakening, the PROMIS Sleep Disturbance-Short Form (Yu et al., 2011), both implemented on the same Android phone.

### Ambulatory session

HR was recorded as beat-to-beat intervals in ms with the RS-800CX (Polar Electro) and the Bodyguard 2 (Firstbeat) HR monitors that have been extensively used for HR recording and analysis (e.g., Porto and Junqueira, 2009). First, each diary entry was labeled in the cardiac data. Then, the 24-h raw beat-to-beat intervals were arranged in 1 and 5-min blocks. HRV was assessed by computing the root mean square of successive beat-to-beat interval differences (RMSSD), which reflects vagal regulation of HR (Task Force, 1996). Outlier and artifact detection as well as HRV analyses were performed using Kubios HRV software (Tarvainen et al., 2014). During waking hours, the 1-min epochs were further averaged based on subject's report of the duration of MW or PC (5-, 10-, 20-, and 30-min). For sleep, the 1-min epochs were further averaged based on self-reports of bed and wake up times.

### Statistical analyses

Data are expressed as means (*SD*). Laboratory data processing and data analyses were performed with Systat 12.0 (Systat Software Inc., Richmond, CA). As the distribution of HRV was non-normal, this variable was log transformed.

To evaluate the effects of socio-demographic factors on the dependent variables, Pearson correlations were performed between BMI, age, years of education, income, physical activity, alcohol, and nicotine consumption, and HR and HRV during waking hours. Gender differences were evaluated by *t*-tests.

Differences in number of episodes of PC, mean HR and HRV during wake and sleep, and socio-personality factors between the two groups were evaluated by *t*-tests and *chi-square* tests.

Random effects regression models are the most appropriate methods of analysis for repeated measures of HR, HRV, and diary variables (Shapiro et al., 2001). The Restricted Maximum Likelihood method was used for model estimation. The covariance model among observations within subject was a random intercept plus Autoregressive model. This approach is particularly suitable, as the periodicity of thoughts, mood, and physiological measurement is likely to be highly heterogeneous, and it also deals with missing values. Differences in sample sizes are due to missing values for some of the variables. Because it models each participant as a random effect, using this procedure accommodates interindividual variation in thoughts-mood–HR/HRV relationships.

The specific cognitive state, group, and group × cognitive state interaction were entered as predictors of each dependent variable: efforts to inhibit, interference with ongoing activities, HR, HRV, and mood. The biobehavioral variables that had a significant relationship with a given dependent variable and the occurrence of stressful episodes were also entered.

Spearman's correlations were performed to test the associations between HRV during PC and (a) somatic complaints during the same day and (b) dispositional characteristics (SRRS, CM, RRS, STAXI, PHQ-9, BDI, and PSWQ). As our participants were not asked to fill out the diary at night, only the association between HRV during sleep and dispositional characteristics (SRRS, CM, RRS, STAXI, PHQ-9, BDI, and PSWQ) was assessed by Spearman's correlations.

To correct for multiple comparisons, Bonferroni corrected *p*-values are presented.

## Results

The only significant associations that emerged between socio-demographic variables and outcome measures were between HR and caffeine consumption (*r* = 0.34; *p* = 0.04) and between HRV and age (*r* = −0.40; *p* = 0.02) and nicotine consumption (*r* = −0.38; *p* = 0.02). Thus, these variables were included in the appropriate regression model. No gender differences emerged, except for a tendency toward lower HRV in women during sleep (*t* = 2.0; *p* = 0.05).

Table 1 shows group differences for the main variables of the study. Compared to healthy controls, MDD participants were older (*t* = 2.2), had higher BMI (*t* = 2.9), had more episodes of PC (*t* = 2.2), and higher scores on the STAXI (*t* = 2.1), BDI (*t* = 6.9), CM (*t* = 2.3), STAI (*t* = 7.1), negative inferential style and hopelessness subscales of the SRRS (*t* = 3.6 and *t* = 6.4, respectively), PHQ-9 (*t* = 9.5), PHQ-15 (*t* = 3.4), RRS (*t* = 8.6), and Loneliness scale (*t* = 4.0). More MDD participants consumed caffeine (χ^2^ = 5.6) and they were less likely to be employed (χ^2^ = 9.2). Compared to controls, MDD participants showed higher HR during wake (*t* = 2.9) and lower HRV during wake and sleep (Table 1; *t* = −2.1 and −4.2, respectively).

**Table 1:** **Group differences for the main variables of the study**.

	**MDD (*n* = 18)**	**HC (*n* = 18)**	***p***
Gender	12F; 6M	11F; 7M	0.73
Age (years)	38.4 (12.1)	30.1 (10.5)	0.03
Education	6C; 1D; 5G; 6P	4C; 2D; 5G; 7P	0.85
Employed	12N; 6Y	3N; 15Y	0.002
BMI (Kg/m^2^)	27.4 (6.7)	22.4 (3.0)	0.01
Ethnicity	3AA; 5AS; 8C; 2L	0AA; 8AS; 5C; 5L	0.13
Smoking	15N; 3Y	18N; 0Y	0.07
Alcohol consumption	13N; 5Y	16N; 2Y	0.21
Caffeine consumption	4N; 14Y	11N; 7Y	0.02
Exercise	5N; 13Y	4N; 14Y	0.70
STAXI	19.5 (5.1)	15.6 (5.8)	0.04
BDI	31.1 (12.2)	6.7 (8.6)	<0.0001
CM	30.5 (22.3)	18.1 (6.5)	0.03
STAI	59.5 (8.8)	35.2 (11.5)	<0.0001
PSWQ	55.0 (14.7)	48.7 (13.3)	0.19
SRRS-PS	356.4 (100.1)	443.7 (154.1)	0.05
SRRS-NIS	586.6 (120.7)	373.9 (218.3)	0.001
SRRS-H	302.7 (109.9)	83.7 (94.5)	<0.0001
PHQ-9	17.9 (6.1)	2.4 (3.2)	<0.0001
RRS	61.4 (8.9)	34.2 (9.9)	<0.0001
Three-Item Loneliness Scale	7.0 (1.8)	4.6 (1.8)	<0.0001
Wake HR (bpm)	85.3 (8.2)	77.6 (7.9)	0.01
Wake RMSSD (ms^2^)	34.9 (16.9)	46.3 (15.3)	0.04
Sleep HR (bpm)	65.7 (8.3)	60.2 (9.4)	0.09
Sleep RMSSD (ms^2^)	41.7 (18.9)	62.8 (33.5)	0.04
Stressful event (n)	2.7 (2.8)	1.3 (1.7)	0.07
Frequency of MW (n)	4.4 (3.2)	7.5 (5.5)	0.05
Frequency of PC (n)	4.5 (2.5)	2.4 (3.3)	0.04
PROMIS	10.4 (3.1)	12.0 (4.3)	0.22
Hours of sleep	6.70 (1.3)	7.26 (1.3)	0.21
Time to sleep	25.18 (23.9)	15.28 (14.4)	0.14
PHQ-15	8.7 (5.6)	3.0 (4.4)	0.002

*M, Males; F, Females; C, College; D, Diploma; G, Graduate; P, Postgraduate; Y, Yes; N, No; AA, African American; AS, Asian; C, Caucasian; L, Latino; SRRS-PS, Problem Solving subscale of the SRRS; SRRS-NIS, Negative Inferential subscale of the SRRS; SRRS-H, Hopelessness subscale of the SRRS*.

**Figure 1:**
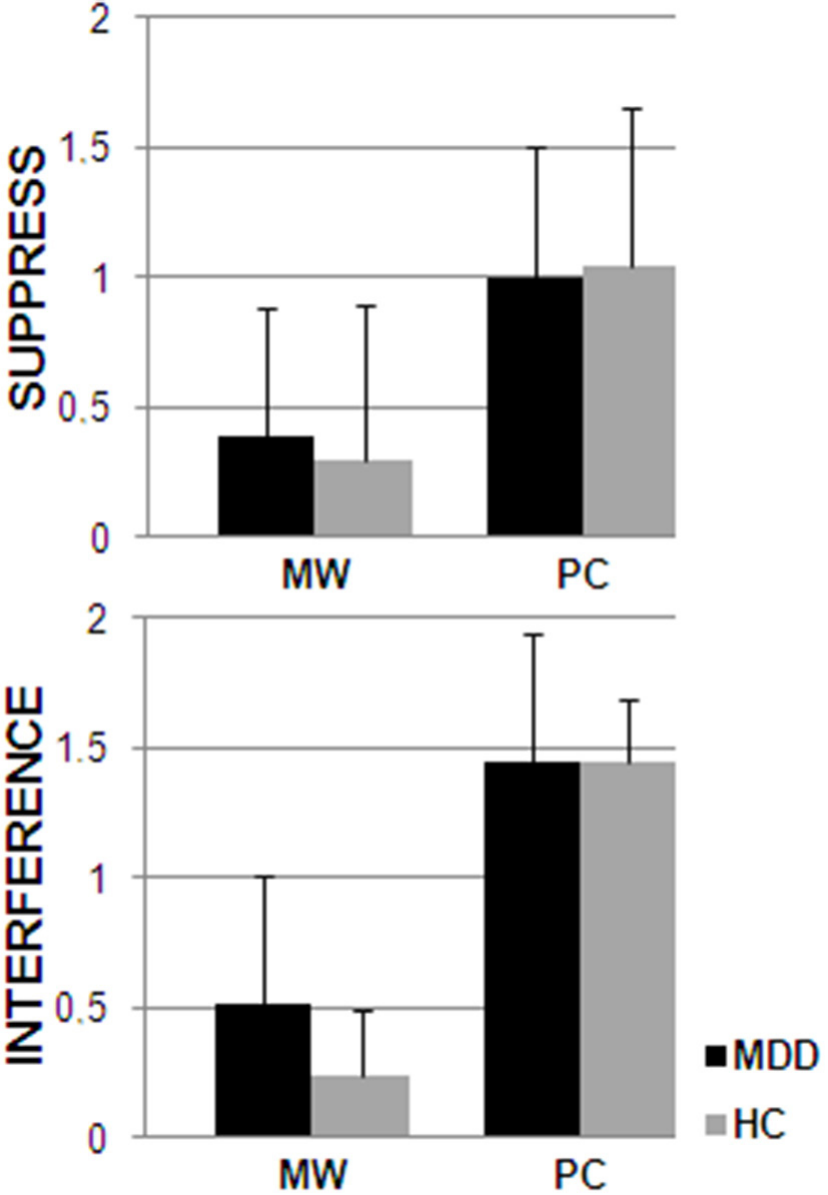
**Answers to the diary questions “Was the thought interfering with what you were doing?” (interference) and “How much were you trying to suppress the thought?” (suppress) for the periods during which participants (MDD and HC) were engaged in MW or PC**.

As to the random regression models, in both groups thoughts required more effort to be inhibited and interfered more with ongoing activities during PC compared to MW (see Figure 1), as indicated by the main effect of cognitive state in these mixed regression models; *F*_(1, 335)_ = 42.67; *p* < 0.0001 and *F*_(1, 335)_ = 124.16; *p* < 0.0001, respectively.

**Figure 2:**
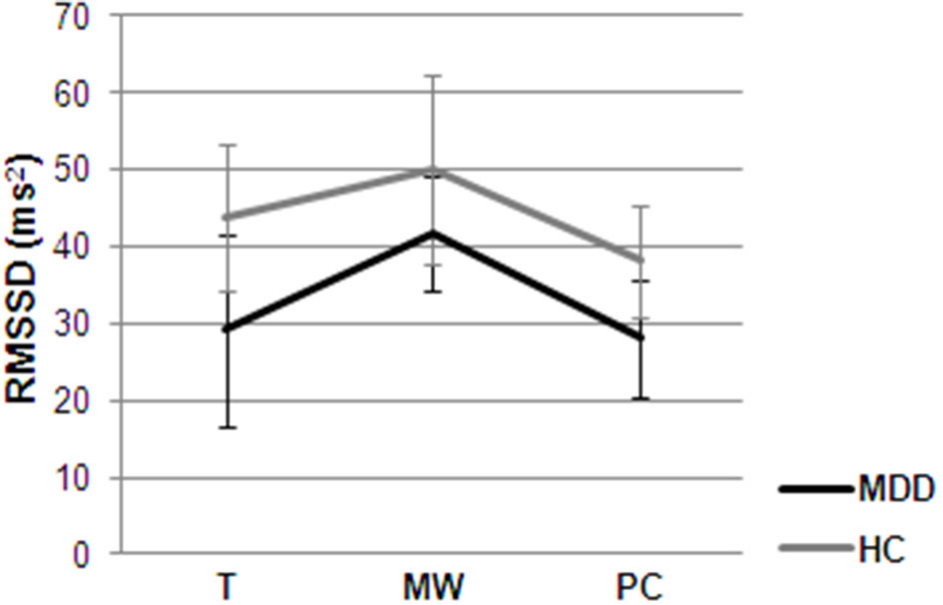
**Mean ambulatory HRV for the periods during which participants (MDD and HC) were engaged in MW, PC or were focused on task (T), adjusted for age and caffeine consumption**.

For the mixed model predicting HR, only group resulted significant as a predictor, with MDD participants having a higher HR compared to controls [*F*_(1, 563)_ = 7.20; *p* = 0.01], irrespective of their ongoing cognitive state. However, cognitive state was a significant predictor of HRV (see Figure 2), showing that PC was associated with a lower HRV compared to MW [*F*_(2, 562)_ = 12.28; *p* < 0.0001], irrespective of group. Moreover, group played a significant role in this model [*F*_(1, 562)_ = 12.68; *p* < 0.0001], with MDD participants being characterized by lower HRV. Age [*F*_(1, 563)_ = 6.6; *p* = 0.01] and nicotine [*F*_(1, 563)_ = 21.05; *p* < 0.0001] were also significant predictors in the model.

**Figure 3:**
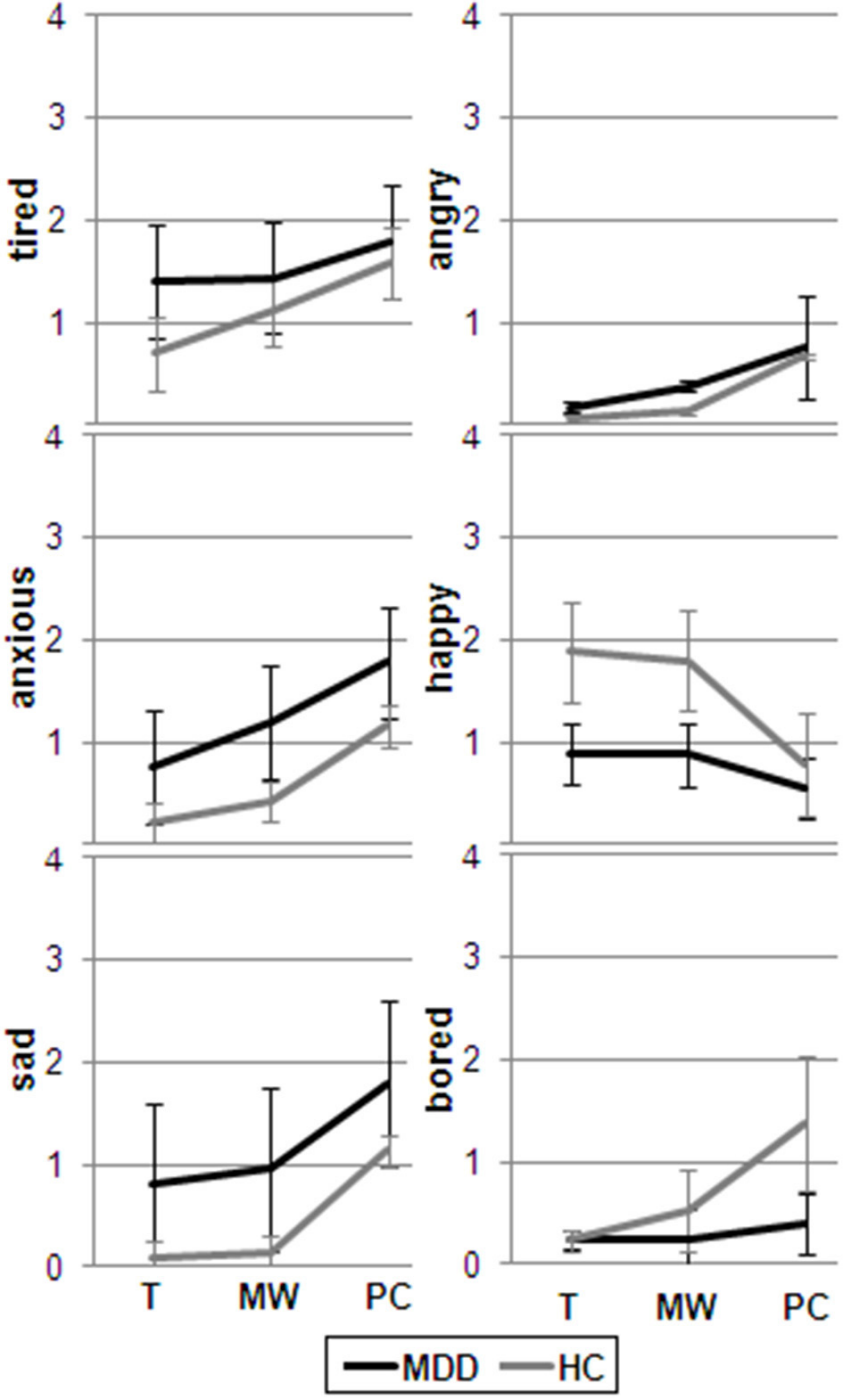
**Mean mood rating for the periods during which participants (MDD and HC) were engaged in MW, PC or were focused on task (T)**.

As to mood (see Figure 3), group was significant in the prediction of being tired [*F*_(1, 564)_ = 4.66; *p* = 0.03], anxious [*F*_(1, 564)_ = 20.75; *p* < 0.0001], sad [*F*_(1, 564)_ = 13.83; *p* < 0.0001], and bored [*F*_(1, 564)_ = 10.57; *p* = 0.001], with MDD participants showing worse mood compared to healthy controls. Cognitive state was significant predictor of being tired [*F*_(2, 564)_ = 14.52; *p* < 0.0001], anxious [*F*_(2, 564)_ = 60.61; *p* < 0.0001], sad [*F*_(2, 564)_ = 66.29; *p* < 0.0001], happy [*F*_(2, 564)_ = 23.14; *p* < 0.0001], angry [*F*_(2, 564)_ = 35.18; *p* < 0.0001], and bored [*F*_(2, 564)_ = 32.47; *p* < 0.0001], with PC being always characterized by worse mood compared to MW and being on task. A significant group x cognitive state interaction emerged for the mixed models predicting happy [*F*_(2, 564)_ = 6.40; *p* = 0.002] and bored [*F*_(2, 564)_ = 17.21; *p* < 0.0001], with healthy controls being less happy and more bored during PC compared to MW and being on task and MDD patients being equally happy and bored irrespective of the ongoing cognitive process.

HRV during PC significantly correlated with somatic complaints on the same day, as assessed by the PHQ-15 (*r*_s_ = −0.31) and with the following dispositional characteristics: hopelessness rumination (SRRS; *r*_s_ = −0.42), trait anxiety (STAI; *R*= −0.40), and depression (PHQ-9; *r*_s_ = −0.39). HRV during sleep was negatively correlated with hopelessness rumination (*r*_s_ = −0.32).

## Discussion

Our main hypothesis that PC would be associated with higher levels of rigidity (cognitive, autonomic, and behavioral) compared to MW was supported by the data. In line with our view that MW is not *per se* maladaptive, only PC was associated with health risk factors (i.e., low HRV) and mood worsening. Several laboratory and ambulatory studies previously found reduced HRV during rumination (e.g., Key et al., 2008; Ottaviani et al., 2008; Ottaviani and Shapiro, 2011) and worry (e.g., Castaneda and Segerstrom, 2004; Hofmann et al., 2005; Delgado et al., 2009; Brosschot et al., 2007; Ottaviani et al., 2014) in healthy participants. On the other hand, psychophysiological studies of MW did not include HRV. To the best of our knowledge, only Ottaviani et al. (2013) directly compared PC and MW in terms of physiological and affective consequences. Here, we replicated and extended our previous findings in two ways: (1) from the laboratory to daily life and (2) from healthy participants to patients with a diagnosis of MDD.

In general, MDD participants had higher HR and lower HRV compared to healthy controls. This confirms previous results (e.g., Koschke et al., 2009) and has important clinical implications considering that (1) low HRV has been associated with increased risk of all-cause mortality (Thayer et al., 2010) and (2) in a recent multi-treatment study, Brunoni et al. (2013) showed that reduced HRV is a trait-marker for MDD and not only a mere consequence of pharmacotherapy. Moreover, PC was characterized by HRV reductions in both MDD and healthy participants compared to MW. This autonomic rigidity was mirrored by cognitive and behavioral inflexibility, as reflected by higher efforts to inhibit the thought and interference with ongoing activities during PC compared to MW. This supports the notion that HRV may index important organism functions associated with adaptability and flexibility, as it indices the degree of functional integration between the ventro-medial prefrontal cortex, brainstem, and peripheral physiology (Thayer and Lane, 2009; Thayer et al., 2012).

Irrespective of the ongoing cognitive process, worse mood was reported by MDD patients compared to controls, except for being angry. This is consistent with a number of ambulatory studies in which MDD patients reported less positive affect and more negative affect than healthy controls (e.g., Peeters et al., 2003b; Bylsma et al., 2011).

Both groups were more anxious, sad, tired, and angry during PC compared to MW and being on task, again replicating previous results (Ottaviani et al., 2013). The effects of PC on mood provide further support to Nolen-Hoeksema's (2004) Response Style Theory which suggests that rumination negatively impacts individuals through the activation of negative thoughts and memories, exacerbating the impact of depressed mood on thinking and increasing the likelihood that individuals will make depressogenic inferences in regard to their current circumstances. In our study, MW did not have different effects in terms of mood worsening compared to being on task, neither in healthy nor in MDD participants. This is in contrast with others' findings (e.g., Killingsworth and Gilbert, 2010), but again, previous studies did not directly compare MW and PC, and probably included both constructs under their definition of MW. In our opinion, it is very important to understand when spontaneous thought is functional (e.g., for creativity and insights) and when it is not, especially for mood disorders. In their recent theoretical model, Tops et al. (2014) suggest that it is possible to make specific neuro-behavioral predictions about adaptive and maladaptive cognitive states based on the theory of predictive and reactive control systems (Tops et al., 2014), further proposing *ad hoc* therapeutic intervention for the so-called ruminating mind. The idea that MW is not *per se* dysfunctional in terms of health and mood consequences has been further supported by a recent longitudinal study conducted by our group (Ottaviani and Couyoumdjian, 2013).

As expected, MDD participants' scores on all dispositional questionnaires were notably higher than scores for healthy controls. Aside from depression, MDD participants felt more lonely, had higher levels of anxiety, anger, hostility, and trait rumination, the latter specifically focused on depressive symptoms and characterized by a negative inferential style and hopelessness. The same results on anxiety, anger, and hostility have been previously reported by our group (Davydov et al., 2007). Other authors confirmed the association between subclinical and clinical depression and loneliness (e.g., Wei et al., 2005), anger (e.g., Besharat et al., 2013), and hostility (e.g., Conrad et al., 2009). The relationship between rumination and MDD is one of the most well-replicated finding in this field (e.g., Nolen-Hoeksema et al., 2008). In fact, rumination has been linked to longer and more severe depression, delayed recovery from depression, increases in suicidal ideation (e.g., Eshun, 2000; Lyubomirsky and Tkach, 2004; Smith et al., 2006), and has also prospectively predicted major depression over a 2.5-year follow up (Nolen-Hoeksema, 2000; Spasojevic and Alloy, 2001).

Compared to healthy controls, MDD participants were not characterized by higher dispositional worry. Even if it seems intuitive that worrisome thoughts about future threats may better correlate with anxiety than depression, this is in disagreement with findings in which worry and rumination did not differentially relate to anxiety or depression (Segerstrom et al., 2000; Hong, 2007). However, inconsistencies may be explained by the fact that previous studies focused on healthy individuals (i.e., students), while our study was conducted on a clinical population. Indeed, the specificity of worry and rumination as vulnerability factors for the development of anxiety and depressive symptoms in children has been more recently reported (Verstraeten et al., 2011).

Surprisingly, no difference emerged between the pathological and healthy samples in terms of quality of sleep. This is contrary to previous studies in which depression and greater levels of intrusive thoughts were associated with both subjective and laboratory-assessed sleep disruptions (Hall et al., 1997; Kecklund and Akerstedt, 2004). We had to rely on subjective reports, which may have limited the reliability of results. However, our more objective marker during sleep (HRV) showed significant differences between the two groups and was negatively correlated with hopelessness rumination. This provides support to the few ambulatory studies that highlighted that -at least at a physiological level—the consequences of PC extend to the following night, as shown by reductions in HRV during sleep (Brosschot et al., 2007; Yoshino and Matsuoka, 2009; Weise et al., 2013).

In agreement with previous data (e.g., Haug et al., 2004), MDD participants reported more somatic symptoms (e.g., headaches, stomach pain) compared to controls during the ambulatory assessment. These cross-sectional findings are experimentally supported by the role of sad mood induction in increasing pain sensitivity in MDD patients (Terhaar et al., 2010). Also, the amount and entity of somatic complaints in our study was negatively correlated with HRV during rumination. The substantial effects of PC on somatic health have been repeatedly demonstrated (reviewed in Verkuil et al., 2010). For example, non-clinical levels of worry were prospectively associated with self-reported health complaints (Brosschot and Van Der Doef, 2006; Jellesma et al., 2009; Verkuil et al., 2012) and even a simple worry intervention resulted effective in reducing health complaints (Brosschot and Van Der Doef, 2006; Jellesma et al., 2009). Here, we replicated these results further supporting the model according to which the physiological activation that characterizes perseverative thoughts may ultimately be responsible for their pathogenic effects on health (i.e., PC Hypothesis, Brosschot et al., 2006, 2010).

Vagally-mediated HRV was also inversely associated with scores on hopelessness rumination, trait anxiety, and depression. In a previous study of ours, similar patterns were found using different measures of vagal functioning such as baroreflex sensitivity and baroreflex effectiveness index in a healthy population (Ottaviani et al., 2009), thus confirming the robustness of findings. Intrusive thoughts affect all individuals, but it is possible that only those with certain dispositional traits may be more likely to experience vagal withdrawal during this cognitive process. Among the examined forms of intrusive thoughts, hopelessness rumination is the most maladaptive as it is associated with the belief that “things will never get better.” It is not therefore surprising that it was the only dispositional type of PC that resulted associated with health consequences (during both wake and sleep). Reduced HRV has been associated with both increased risk of cardiovascular disease (see Thayer et al., 2010 for a review), MDD (e.g., Koschke et al., 2009), and PC (e.g., Ottaviani et al., 2008, 2014; Ottaviani and Shapiro, 2011). Prolonged autonomic dysregulation has been proposed as one potential mechanism linking depression, stress, and cardiovascular disorder (Larsen and Christenfeld, 2009; Gerin et al., 2012). Longitudinal studies are needed to disentangle causal relationships.

A limitation of the present study is the failure to examine the duration of physiological activation after PC, as there is evidence that prolonged physiological activity predicts pathogenic states and organic disease far beyond the immediate cardiovascular reaction, and prolonged activation during PC is perhaps only a part of the total amount of potentially pathogenic prolonged physiological activation (Brosschot et al., 2010). Second, the sample size was relatively small, due to difficulties in MDD participant recruitment. To overcome this limit, repeated measurements and mixed models were used in the main analyses. Third, there was an unequal gender distribution in our sample. Although this reflects the fact that MDD is more frequent in women compared to men, this may have biased some of the results, such as the absence of gender differences in HRV levels. Despite limitations of this study, we believe that we have provided implicative data for future studies regarding the still obscure link between depression and cardiovascular risk, suggesting that—if prolonged—the reduced HRV that characterizes PC may play a role in this relationship.

### Conflict of interest statement

The authors declare that the research was conducted in the absence of any commercial or financial relationships that could be construed as a potential conflict of interest.

## References

[B1] Aan het Rot M. Hogenelst K. Schoevers R. A. (2012). Mood disorders in everyday life: a systematic review of experience sampling and ecological momentary assessment studies. Clin. Psychol. Rev. 32, 510–523. 10.1016/j.cpr.2012.05.007 22721999

[B2] Beck A. T. Steer R. A. Brown G. K. (1996). Manual for the Beck Depression Inventory-II. San Antonio, TX: Psychological Corporation.

[B3] Besharat M. A. Nia M. E. Farahani H. (2013). Anger and major depressive disorder: the mediating role of emotion regulation and anger rumination. Asian J. Psychiatr. 6, 35–41. 10.1016/j.ajp.2012.07.013 23380315

[B4] Brosschot J. F. Van Der Doef M. (2006). Daily worrying and somatic health complaints: testing the effectiveness of a simple worry reduction intervention. Psychol. Health 21, 19–31 10.1080/14768320500105346

[B5] Brosschot J. F. Gerin W. Thayer J. F. (2006). The perseverative cognition hypothesis: a review of worry, prolonged stress-related physiological activation, and health. J. Psychosom. Res. 60, 113–124. 10.1016/j.jpsychores.2005.06.074 16439263

[B6] Brosschot J. F. Van Dijk E. Thayer J. F. (2007). Daily worry is related to low heart rate variability during waking and the subsequent nocturnal sleep period. Int. J. Psychophysiol. 63, 39–47. 10.1016/j.ijpsycho.2006.07.016 17020787

[B7] Brosschot J. F. Verkuil B. Thayer J. F. (2010). Conscious and unconscious perseverative cognition:is a large part of prolonged physiological activity due to unconscious stress? J. Psychosom. Res. 69, 407–416. 10.1016/j.jpsychores.2010.02.002 20846542

[B8] Brunoni A. R. Kemp A. H. Dantas E. M. Goulart A. C. Nunes M. A. Boggio P. S.. (2013). Heart rate variability is a trait marker of major depressive disorder: evidence from the sertraline vs. electric current therapy to treat depression clinical study. Int. J. Neuropsychopharmacol. 16, 1937–1949. 10.1017/S1461145713000497 23759172

[B9] Bylsma L. M. Taylor-Clift A. Rottenberg J. (2011). Emotional reactivity to daily events in major and minor depression. J. Abnorm. Psychol. 120, 155–167. 10.1037/a0021662 21319928

[B10] Carriere J. S. Seli P. Smilek D. (2013). Wandering in both mind and body: individual differences in mind wandering and inattention predict fidgeting. Can. J. Exp. Psychol. 67, 19–31. 10.1037/a0031438 23458548

[B11] Castaneda J. O. Segerstrom S. C. (2004). Effect of stimulus type and worry on physiological response to fear. J. Anxiety Disord. 18, 809–823. 10.1016/j.janxdis.2003.10.003 15474854

[B12] Chambers A. S. Allen J. J. (2002). Vagal tone as an indicator of treatment response in major depression. Psychophysiology 39, 861–864. 10.1111/1469-8986.3960861 12462513

[B13] Conrad R. Wegener I. Imbierowicz K. Liedtke R. Geiser F. (2009). Alexithymia, temperament and character as predictors of psychopathology in patients with major depression. Psychiatry Res. 165, 137–144. 10.1016/j.psychres.2007.10.013 19084277

[B14] Cook W. W. Medley D. M. (1954). Proposed hostility and pharisaic-virtue scales for the MMPI. J. Appl. Psychol. 238, 414–418 10.1037/h0060667

[B15] Davydov D. M. Shapiro D. Cook I. A. Goldstein I. (2007). Baroreflex mechanisms in major depression. Prog. Neuropsychopharmacol. Biol. Psychiatry 31, 164–177. 10.1016/j.pnpbp.2006.08.015 17011098

[B16] Delgado L. C. Guerra P. Perakakis P. Mata J. L. Perez M. N. Vila J. (2009). Psychophysiological correlates of chronic worry: cued versus non-cued fear reaction. Int. J. Psychophysiol. 74, 280–287. 10.1016/j.ijpsycho.2009.10.007 19819267

[B17] Eshun S. (2000). Role of gender and rumination in suicide ideation: a comparison of college samples from Ghana and the United States. Cross Cult. Res. 34, 250–263 10.1177/106939710003400303

[B18] Gerin W. Zawadzki M. J. Brosschot J. F. Thayer J. F. Christenfeld N. J. Campbell T. S.. (2012). Rumination as a mediator of chronic stress effects on hypertension: a causal model. Int. J. Hypertens. 2012:453465. 10.1155/2012/453465 22518285 PMC3296188

[B19] Hall M. Buysse D. J. Dew M. A. Prigerson H. G. Kupfer D. J. Reynolds C. F. III. (1997). Intrusive thoughts and avoidance behaviors are associated with sleep disturbances in bereavement-related depression. Depress. Anxiety 6, 106–112. 10.1002/(SICI)1520-6394(1997)6:3<106::AID-DA3>3.0.CO;2-B 9442984

[B20] Haug T. T. Mykletun A. Dahl A. A. (2004). The association between anxiety, depression, and somatic symptoms in a large population: the HUNT-II study. Psychosom. Med. 66, 845–851. 10.1097/01.psy.0000145823.85658.0c 15564348

[B21] Hofmann S. G. Moscovitch D. A. Litz B. T. Kim H. Davis L. L. Pizzagalli D. A. (2005). The worried mind: autonomic and prefrontal activation during worrying. Emotion 5, 464–475. 10.1037/1528-3542.5.4.464 16366750

[B22] Hong R. Y. (2007). Worry and rumination: differential associations with anxious and depressive symptoms and coping behavior. Cog. Ther. Res. 45, 277–290. 10.1016/j.brat.2006.03.006 16635479

[B22a] Huffziger S. Ebner-Priemer U. Zamoscik V. Reinhard I. Kirsch P. Kuehner C. (2013). Effects of mood and rumination on cortisol levels in daily life: an ambulatory assessment study in remitted depressed patients and healthy controls. Psychoneuroendocrinology 38, 2258–2267. 10.1016/j.psyneuen.2013.04.014 23684479

[B23] Hughes M. E. Waite L. J. Hawkley L. C. Cacioppo J. T. (2004). A short scale for measuring loneliness in large surveys. Res. Aging 26, 655–672. 10.1177/0164027504268574 18504506 PMC2394670

[B24] Jain F. A. Cook I. A. Leuchter A. F. Hunter A. M. Tartter M. Davydov D. M.. (2014). Heart rate variability and treatment outcome in major depression: a pilot study. Int. J. Psychophys. 93, 204–210. 10.1016/j.ijpsycho.2014.04.006 24769434

[B25] Jellesma F. C. Verkuil B. Brosschot J. F. (2009). Postponing worrisome thoughts in children: the effects of a postponement intervention on perseverative thoughts, emotions and somatic complaints. Soc. Sci. Med. 69, 160–164. 10.1016/j.socscimed.2009.04.031 19520470

[B26] Karavidas M. K. Lehrer P. M. Vaschillo E. Vaschillo B. Marin H. Buyske S.. (2007). Preliminary results of an open label study of heart rate variability biofeedback for the treatment of major depression. Appl. Psychophysiol. Biofeedback 32, 19–30. 10.1007/s10484-006-9029-z 17333315

[B27] Kecklund G. Akerstedt T. (2004). Apprehension of the subsequent working day is associated with a low amount of slow wave sleep. Biol. Psychol. 66, 169–176. 10.1016/j.biopsycho.2003.10.004 15041138

[B28] Key B. L. Campbell T. S. Bacon S. L. Gerin W. (2008). The influence of trait and state rumination on cardiovascular recovery from a negative emotional stressor. J. Behav. Med. 31, 237–248. 10.1007/s10865-008-9152-9 18350377

[B29] Killingsworth M. A. Gilbert D. T. (2010). Awandering mind is an unhappy mind. Science 330, 932. 10.1126/science.1192439 21071660

[B30] Koschke M. Boettger M. K. Schulz S. Berger S. Terhaar J. Voss A.. (2009). Autonomy of autonomic dysfunction in major depression. Psychosom. Med. 71, 852–860. 10.1097/PSY.0b013e3181b8bb7a 19779146

[B31] Kroenke K. Spitzer R. L. Williams J. B. (2001). The PHQ-9: validity of a brief depression severity measure. J. Gen. Intern. Med. 16, 606–613. 10.1046/j.1525-1497.2001.016009606.x 11556941 PMC1495268

[B32] Kroenke K. Spitzer R. L. Williams J. B. (2002). The PHQ-15: validity of a new measure for evaluating the severity of somatic symptoms. Psychosom. Med. 64, 258–266. 10.1097/00006842-200203000-00008 11914441

[B33] Larsen B. A. Christenfeld N. J. (2009). Cardiovascular disease and psychiatric comorbidity: the potential role of perseverative cognition. Cardiovasc. Psychiatry Neurol. 2009:791017. 10.1155/2009/791017 20029626 PMC2790803

[B34] Lyubomirsky S. Tkach C. (2004). The consequences of dysphoric rumination, in Rumination: Nature, Theory, and Treatment of Negative Thinking in Depression, eds Papageorgiou C. Wells A. (Chichester: John Wiley & Sons), 21–41.

[B35] Marchetti I. van de Putte E. Koster E. H. W. (2014). Self-generated thoughts and depression: from daydreaming to depressive symptoms. Front. Hum. Neurosci. 8:131. 10.3389/fnhum.2014.00131 24672458 PMC3957030

[B36] McVay J. C. Kane M. J. Kwapil T. R. (2009). Tracking the train of thought from the laboratory into everyday life: an experience-sampling study of mind-wandering across controlled and ecological contexts. Psychon. Bull. Rev. 16, 857–863. 10.3758/PBR.16.5.857 19815789 PMC2760023

[B37] Meyer T. J. Miller M. L. Metzger R. L. Borkovec T. D. (1990). Development and validation of the Penn State Worry Questionnaire. Behav. Res. Ther. 28, 487–495. 10.1016/0005-7967(90)90135-6 2076086

[B38] Murphy F. Macpherson K. Jeyabalasingham T. Manly T. Dunn B. (2013). Modulating mind-wandering in dysphoria. Front. Psychol. 4:888. 10.3389/fpsyg.2013.00888 24348442 PMC3841720

[B39] Nolen-Hoeksema S. (2000). The role of rumination in depressive disorders and mixed anxiety/depressive symptoms. J. Abnorm. Psychol. 109, 504–511. 10.1037/0021-843X.109.3.504 11016119

[B40] Nolen-Hoeksema S. (2004). The response styles theory, in Depressive Rumination: Nature, Theory, and Treatment, eds Papageorgiou C. Wells A. (New York, NY: Wiley), 107–124.

[B41] Nolen-Hoeksema S. Morrow J. (1991). A prospective study of depression and posttraumatic stress symptoms after a natural disaster: the 1989 Loma Prieta Earthquake. J. Pers. Soc. Psychol. 61, 115–121. 10.1037/0022-3514.61.1.115 1890582

[B42] Nolen-Hoeksema S. Wisco B. E. Lyubomirsky S. (2008). Rethinking rumination. Perspect. Psychol. Sci. 3, 400–424 10.1111/j.1745-6924.2008.00088.x 26158958

[B43] Onraedt T. Koster E. H. (2014). Training working memory to reduce rumination. PLoS ONE 9:e90632. 10.1371/journal.pone.0090632 24595102 PMC3940909

[B44] Ottaviani C. Couyoumdjian A. (2013). Pros and cons of a wandering mind: a prospective study. Front. Psychol. 4:524. 10.3389/fpsyg.2013.00524 23966964 PMC3743222

[B45] Ottaviani C. Shapiro D. (2011). Do we need a stressor to be stressed? Insights from cardiac regulation. Jpn. Psychol. Res. 53, 155–162 10.1111/j.1468-5884.2011.00462.x

[B46] Ottaviani C. Borlimi R. Brighetti G. Caselli G. Favaretto E. Giardini I.. (2014). Worry as an adaptive avoidance strategy in healthy controls but not in pathological worriers. Int. J. Psychophys. 93, 349–355. 10.1016/j.ijpsycho.2014.05.010 24873888

[B47] Ottaviani C. Shapiro D. Couyoumdjian A. (2013). Flexibility as the key for somatic health: from mind wandering to perseverative cognition. Biol. Psychol. 94, 38–43. 10.1016/j.biopsycho.2013.05.003 23680439

[B48] Ottaviani C. Shapiro D. Fitzgerald L. (2011). Rumination in the lab: what happens when you go back to everyday life? Psychophysiology 48, 453–461. 10.1111/j.1469-8986.2010.01122.x 20846182

[B49] Ottaviani C. Shapiro D. Davydov D. M. Goldstein I. B. (2008). Autonomic stress response modes and ambulatory heart rate level and variability. J. Psychophys. 22, 28–40 10.1027/0269-8803.22.1.28

[B50] Ottaviani C. Shapiro D. Davydov D. M. Goldstein I. B. Mills P. J. (2009). The autonomic phenotype of rumination. Int. J. Psychophys. 72, 267–275. 10.1016/j.ijpsycho.2008.12.014 19272312

[B51] Peeters F. Nicolson N. A. Berkhof J. (2003a). Cortisol responses to daily events in major depressive disorder. Psychosom. Med. 65, 836–841. 10.1097/01.PSY.0000088594.17747.2E 14508029

[B52] Peeters F. Nicolson N. A. Berkhof J. (2004). Levels and variability of daily life cortisol secretion in major depression. Psychiatry Res. 126, 1–13. 10.1016/j.psychres.2003.12.010 15081622

[B53] Peeters F. Nicolson N. A. Berkhof J. Delespaul P. deVries M. (2003b). Effects of daily events on mood states in major depressive disorder. J. Abnorm. Psychol. 112, 203–211. 10.1037/0021-843X.112.2.203 12784829

[B54] Pieper S. Brosschot J. F. van der Leeden R. Thayer J. F. (2007). Cardiac effects of momentary assessed worry episodes and stressful events. Psychosom. Med. 69, 901–909. 10.1097/PSY.0b013e31815a9230 17991822

[B55] Pieper S. Brosschot J. F. van der Leeden R. Thayer J. F. (2010). Prolonged cardiac effects of momentary assessed stressful events and worry episodes. Psychosom. Med. 72, 570–577. 10.1097/PSY.0b013e3181dbc0e9 20410249

[B56] Poerio G. L. Totterdell P. Miles E. (2013). Mind-wandering and negative mood: does one thing really lead to another? Conscious. Cogn. 22, 1412–1421. 10.1016/j.concog.2013.09.012 24149091

[B57] Porto L. G. Junqueira L. F. Jr. (2009). Comparison of time-domain short-term heart interval variability analysis using a wrist-worn heart rate monitor and the conventional electrocardiogram. Pacing Clin. Electrophysiol. 32, 43–51. 10.1111/j.1540-8159.2009.02175.x 19140912

[B58] Putnam K. M. McSweeney L. B. (2008). Depressive symptoms and baseline prefrontal EEG alpha activity: a study utilizing ecological momentary assessment. Biol. Psychol. 77, 237–240. 10.1016/j.biopsycho.2007.10.010 18079035

[B59] Robinson M. S. Alloy L. B. (2003). Negative cognitive styles and stress-reactive rumination interact to predict depression: a prospective study. Cogn. Ther. Res. 27, 275–292 10.1023/A:1023914416469

[B60] Segerstrom S. C. Tsao J. C. Alden L. E. Craske M. G. (2000). Worry and rumination: repetitive thought as a concomitant and predictor of negative mood. Cogn. Ther. Res. 24, 671–688 10.1023/A:1005587311498

[B60a] Shapiro D. Jamner L. D. Goldstein I. B. Delfino R. J. (2001). Striking a chord: moods, blood pressure, and heart rate in everyday life. Psychophysiology 38, 197–204. 11347865

[B61] Siegle G. J. Price R. B. Jones N. P. Ghinassi F. Painter T. Thase M. E. (2014). You gotta work at it: pupillary indices of task focus are prognostic for response to a neurocognitive. Clin. Psychol. Sci. 2, 455–471 10.1177/2167702614536160

[B62] Slatcher R. B. Robles T. F. Repetti R. L. Fellows M. D. (2010). Momentary work worries, marital disclosure, and salivary cortisol among parents of young children. Psychosom. Med. 72, 887–896. 10.1097/PSY.0b013e3181f60fcc 20841560 PMC2978267

[B63] Smallwood J. Schooler J. W. (2014). The science of mind wandering: empirically navigating the stream of consciousness. Annu. Rev. Psychol. [Epub ahead of print]. 10.1146/annurev-psych-010814-015331 25293689

[B64] Smallwood J. Fitzgerald A. Miles L. K. Phillips L. H. (2009). Shifting moods, wandering minds: negative moods lead the mind to wander. Emotion 9, 271–276. 10.1037/a0014855 19348539

[B65] Smallwood J. O'Connor R. C. Sudberry M. V. Obonsawin M. (2007). Mind-wandering and dysphoria. Cogn. Emot. 21, 816–842 10.1080/02699930600911531

[B66] Smith J. M. Alloy L. B. Abramson L. Y. (2006). Cognitive vulnerability to depression, rumination, hopelessness, and suicidal ideation: multiple pathways to self-injurious thinking. Suicide Life Threat. Behav. 36, 445–456. 10.1521/suli.2006.36.4.443 16978098

[B67] Song X. Wang X. (2012). Mind wandering in Chinese daily lives–an experience sampling study. PLoS ONE 7:e44423. 10.1371/journal.pone.0044423 22957071 PMC3434139

[B68] Spasojevic J. Alloy L. B. (2001). Rumination as a common mechanism relating depressive risk factors to depression. Emotion 1, 25–37. 10.1037/1528-3542.1.1.25 12894809

[B69] Spielberger C. D. Gorsuch R. L. Lushene R. E. (1970). STAI Manual. Palo Alto, CA: Consulting Psychologists Press.

[B70] Spielberger C. D. Johnson E. H. Russell S. F. Crane R. J. Jacobs G. A. Worden T. J. (1985). The experience and expression of anger: construction and validation of an anger expression scale, in Anger and Hostility in Cardiovascular And Behavioral Disorders, eds Chesney M. A. Rosenman R. H. (Washington, DC: Hemisphere), 5–30.

[B71] Stawarczyk D. Majerus S. D'Argembeau A. (2013). Concern-induced negative affect is associated with the occurrence and content of mind-wandering. Conscious. Cogn. 22, 442–448. 10.1016/j.concog.2013.01.012 23466878

[B72] Task Force of the European Society of Cardiology and the North American Society of Pacing and Electrophysiology (1996). Heart rate variability: standards of measurement, physiological interpretation, and clinical use. Circulation 93, 1043–1065. 10.1161/01.CIR.93.5.1043 8598068

[B73] Tarvainen M. P. Niskanen J. P. Lipponen J. A. Ranta-Aho P. O. Karjalainen P. A. (2014). Kubios HRV–heart rate variability analysis software. Comput. Methods Programs Biomed. 113, 210–220. 10.1016/j.cmpb.2013.07.024 24054542

[B74] Teasdale J. D. (1999). Metacognition, mindfulness and the modification of mood disorders. Clin. Psychol. Psychother. 6, 146–155.

[B75] Terhaar J. Boettger M. K. Schwier C. Wagner G. Israel A. K. Bär K. J. (2010). Increased sensitivity to heat pain after sad mood induction in female patients with major depression. Eur. J. Pain 14, 559–563. 10.1016/j.ejpain.2009.09.004 19837623

[B76] Thayer J. F. Åhs F. Fredrikson M. Sollers J. J III. Wager T. D. (2012). A meta-analysis of heart rate variability and neuroimaging studies: Implications for heart rate variability as a marker of stress and health. Neurosci. Biobehav. Rev. 36, 747–756. 10.1016/j.neubiorev.2011.11.009 22178086

[B77] Thayer J. F. Lane R. D. (2009). Claude Bernard and the heart-brain connection: further elaboration of a model of neurovisceral integration. Neurosci. Biobehav. Rev. 33, 81–88. 10.1016/j.neubiorev.2008.08.004 18771686

[B78] Thayer J. F. Yamamoto S. S. Brosschot J. F. (2010). The relationship of autonomic imbalance, heart rate variability and cardiovascular disease risk factors. Int. J. Cardiol. 141, 122–131. 10.1016/j.ijcard.2009.09.543 19910061

[B79] Tops M. Boksem M. A. S. Quirin M. IJzerman H. Koole S. L. (2014). Internally-directed cognition and mindfulness: an integrative perspective derived from predictive and reactive control systems theory. Front. Psychol. 5:429. 10.3389/fpsyg.2014.00429 24904455 PMC4033157

[B80] Udupa K. Sathyaprabha T. N. Thirthalli J. Kishore K. R. Lavekar G. S. Raju T. R.. (2007). Alteration of cardiac autonomic functions in patients with major depression: a study using heart rate variability measures. J. Affect. Disord. 100, 137–141. 10.1016/j.jad.2006.10.007 17113650

[B81] Unsworth N. McMillan B. D. Brewer G. A. Spillers G. J. (2012). Everyday attention failures: an individual differences investigation. J. Exp. Psychol. Learn. Mem. Cogn. 38, 1765–1772. 10.1037/a0028075 22468805

[B82] Verkuil B. Brosschot J. F. Gebhardt W. A. Thayer J. F. (2010). When worries make you sick: a review of perseverative cognition, the default stress response and somatic health. J. Exp. Psychopathol. 1, 87–118 10.5127/jep.009110

[B83] Verkuil B. Brosschot J. F. Meerman E. E. Thayer J. F. (2012). Effects of momentary assessed stressful events and worry episodes on somatic health complaints. Psychol. Health 27, 141–158. 10.1080/08870441003653470 21038174

[B84] Verstraeten K. Bijttebier P. Vasey M. W. Raes F. (2011). Specificity of worry and rumination in the development of anxiety and depressive symptoms in children. Br. J. Clin. Psychol. 50, 364–378. 10.1348/014466510X532715 22003947

[B85] Wang Y. Zhao X. O'Neil A. Turner A. Liu X. Berk M. (2013). Altered cardiac autonomic nervous function in depression. BMC Psychiatry 13:187. 10.1186/1471-244X-13-187 23842138 PMC3710510

[B86] Watkins E. Moulds M. Mackintosh B. (2005). Comparisons between rumination and worry in a non-clinical population. Behav. Res. Ther. 43, 1577–1585. 10.1016/j.brat.2004.11.008 16239152

[B87] Wei M. Shaffer P. A. Young S. K. Zakalik R. A. (2005). Adult attachment, shame, depression, and loneliness: the mediation role of basic psychological needs satisfaction. J. Couns. Psychol. 52, 591–601 10.1037/0022-0167.52.4.591

[B88] Weise S. Ong J. Tesler N. A. Kim S. Roth W. T. (2013). Worried sleep: 24-h monitoring in high and low worriers. Biol. Psychol. 94, 61–70. 10.1016/j.biopsycho.2013.04.009 23643927

[B89] Williams J. M. G. Kuyken W. (2012). Mindfulness-based cognitive therapy: a promising new approach to preventing depressive relapse. Br. J. Psychiatry 200, 359–360. 10.1192/bjp.bp.111.104745 22550328

[B90] Yoshino K. Matsuoka K. (2009). Effect of mood during daily life on autonomic nervous activity balance during subsequent sleep. Auton. Neurosci. 150, 147–149. 10.1016/j.autneu.2009.03.013 19403341

[B91] Yu L. Buysse D. J. Germain A. Moul D. E. Stover A. Dodds N. E.. (2011). Development of short forms from the PROMIS™ sleep disturbance and sleep-related impairment item banks. Behav. Sleep Med. 10, 6–24. 10.1080/15402002.2012.636266 22250775 PMC3261577

